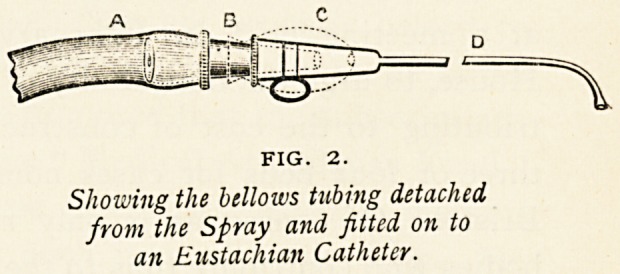# Notes on New Drugs and Preparations for the Sick

**Published:** 1902-03

**Authors:** 


### Notes on New Drugs and Preparations for the Sick.

**Rogers' Sprays.**—H. E. Matthews, Clifton [Agent].—Personal experience with the sprays designed by Rogers has proved their convenience, cleanliness, and reliability. The No. 1 spray is very serviceable in general practice, being equally applicable for aqueous solutions to nose or pharynx. For the larynx and naso-pharynx the adjustable aquol spray is excellent, as it can be manipulated by the right hand alone, leaving the left hand free. The pocket atomiser and nasal oil atomiser, too, are to be commended for simplicity and for throwing a fine spray of oily solutions.

FIG. 1.

*Rogers' No. 1 Spray.*

FIG. 2.

*Showing the bellows tubing detached from the Spray and fitted on to an Eustachian Catheter.*

**Cascara Sagrada (Hawley).**—Evans, Lescher & Webb, London.—The value of cascara in various forms for the treatment of constipation is widely known and appreciated, and of the many excellent preparations on the market we know of none better and more pleasant in administration than these capsules.

---

**Savaresse's Membranous Capsules.**—EVANS, LESCHER & WEBB, London.—To disguise the taste of nauseous remedies, such as Sandal Wood Oil or Balsam of Copaiba, gelatine capsules have been largely used. But inasmuch as the gelatine covering is soluble in the stomach, the capsules burst there, and eructations and nausea are prone to cause much discomfort to the patient. These Membranous Capsules have been introduced to obviate these disadvantages, and as they are almost insoluble till they have passed the stomach and have reached the small intestine, they are a great improvement on gelatine capsules and a distinct advance in pharmacy of considerable practical value.

---

**Merck's Pure Glycerinated Calf Vaccine Lymph** (Tube 351311).—16 Jewry Street, London, E.C.—It is at the present time of the highest importance that we should have absolutely reliable vaccine lymph. Complaints of inefficiency have been frequent of late, but this preparation has been made for many years under the stringent safeguards which are officially demanded in Germany, and is shown by clinical results and bacteriological examination to be one of the best and most reliable.

Dr. J. O. Symes has made an examination of a sample, and he reports that "in examining the preparation for extraneous organisms it maintained a high standard of purity." The notice which accompanies each tube, stating the length of time which it may be kept without losing its potency, is calculated to prevent vexatious disappointments from unsuccessful vaccination. We are informed that this lymph gives results clinically which cannot well be surpassed.

## Figures and Tables

**Fig. 1. f1:**
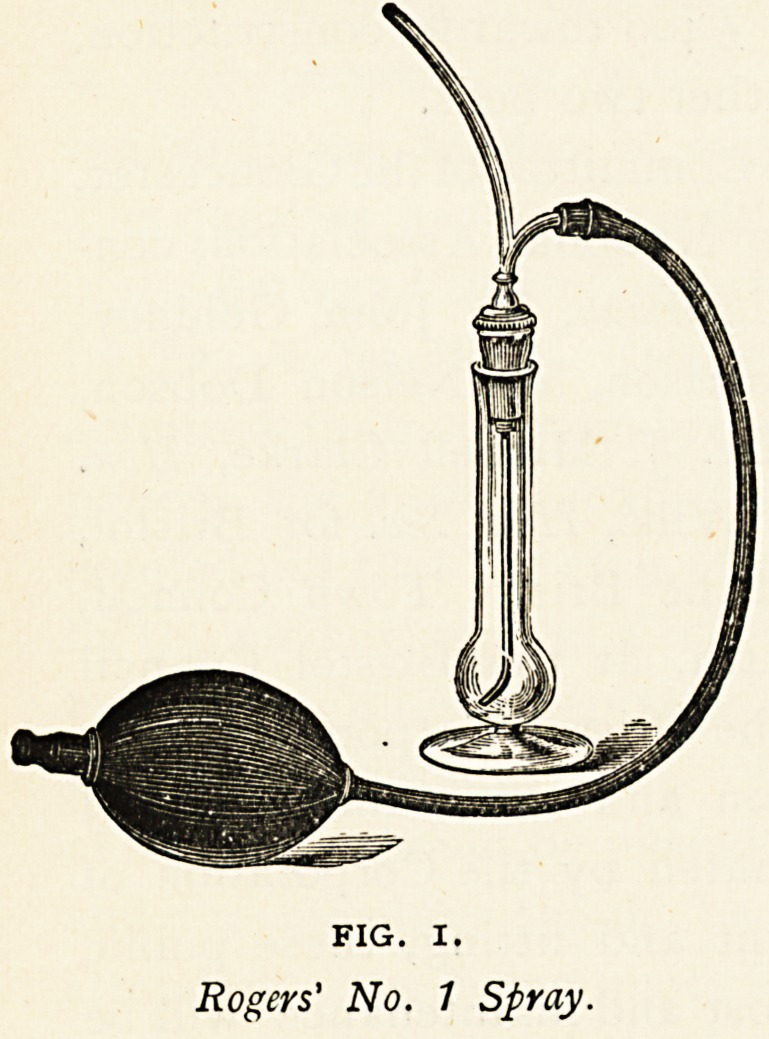


**Fig. 2. f2:**